# Extreme precipitation and emergency room visits for influenza in Massachusetts: a case-crossover analysis

**DOI:** 10.1186/s12940-017-0312-7

**Published:** 2017-10-17

**Authors:** Genee S. Smith, Kyle P. Messier, James L. Crooks, Timothy J. Wade, Cynthia J. Lin, Elizabeth D. Hilborn

**Affiliations:** 1 Oak Ridge Institute for Science and Education, Oak Ridge National Laboratory, Oak Ridge, TN USA; 2 University of North Carolina, Gillings School of Global Public Health, Chapel Hill, NC USA; 3 National Jewish Health, Division of Biostatistics and Bioinformatics, Denver, CO USA; 4 Department of Epidemiology, Colorado School of Public Health, Aurora, CO USA; 5 United States Environmental Protection Agency, Office of Research and Development, National Health and Environmental Effects Research Laboratory, Environmental Public Health Division, MD 58A, Research Triangle Park, Chapel Hill, NC 27711 USA

**Keywords:** Climate, Precipitation, Rainfall, Extreme weather, Influenza, Flu

## Abstract

**Background:**

Influenza peaks during the wintertime in temperate regions and during the annual rainy season in tropical regions – however reasons for the observed differences in disease ecology are poorly understood. We hypothesize that episodes of extreme precipitation also result in increased influenza in the Northeastern United States, but this association is not readily apparent, as no defined ‘rainy season’ occurs. Our objective was to evaluate the association between extreme precipitation (≥ 99th percentile) events and risk of emergency room (ER) visit for influenza in Massachusetts during 2002–2008.

**Methods:**

A case-crossover analysis of extreme precipitation events and influenza ER visits was conducted using hospital administrative data including patient town of residence, date of visit, age, sex, and associated diagnostic codes. Daily precipitation estimates were generated for each town based upon data from the National Oceanic and Atmospheric Administration. Odds ratio (OR) and 95% confidence intervals (CI) for associations between extreme precipitation and ER visits for influenza were estimated using conditional logistic regression.

**Results:**

Extreme precipitation events were associated with an OR = 1.23 (95%CI: 1.16, 1.30) for ER visits for influenza at lag days 0–6. There was significant effect modification by race, with the strongest association observed among Blacks (OR = 1.48 (1.30, 1.68)).

**Conclusions:**

We observed a positive association between extreme precipitation events and ER visits for influenza, particularly among Blacks. Our results suggest that influenza is associated with extreme precipitation in a temperate area; this association could be a result of disease ecology, behavioral changes such as indoor crowding, or both. Extreme precipitation events are expected to increase in the Northeastern United States as climate change progresses. Additional research exploring the basis of this association can inform potential interventions for extreme weather events and influenza transmission.

## Background

Worldwide, influenza is a major, and often preventable, cause of morbidity and mortality. The global burden of influenza infections results in up to five million cases of severe illness and 500,000 deaths annually [1]. The primary modes of transmission for most acute respiratory pathogens, including influenza, are through the respiratory route via large respiratory droplets, small droplet nuclei and via contact with contaminated fomites [2]. Influenza transmission via aerosols and influenza virus survival are both reported to be enhanced in low humidity environments such as those found in heated indoor environments [3–5].

The incidence of influenza infections outside of pandemic years tends to exhibit a seasonal pattern. In temperate regions like the Northeastern U.S., influenza has a clear winter-time peak that corresponds with the cold, winter months [6–8]. However, in tropical climates, acute respiratory infections, including influenza, peak during the rainy season [9–14]. In a retrospective survey of laboratory virus isolates in Singapore, Chew found influenza B isolations to be positively associated with daily rainfall [11]. A seasonal analysis of influenza surveillance conducted in India from 1978 to 1990 observed a positive association (*r* = 0.697, *p* < 0.05) with rainfall [13]. In later years (2007–2008), a 24 month study comparing throat and nasal swabs from young children also in India found a strong positive correlation between influenza A virus and rainfall (*r* = 0.901, *p* < 0.0001) as well [10]. Additional studies observed positive, but not statistically significant, associations between rainfall and influenza in infants and children [12, 14].

Respiratory transmission of influenza virus via droplets and contact transmission via contaminated fomites are facilitated by combining both infected and susceptible individuals together in close proximity [8, 9]. Several investigations have reported that crowded conditions increase risk of influenza and other respiratory illnesses [7, 9, 15]. Extreme rainfall such as that observed during the rainy season in tropical climates may also result in indoor congregation and crowding and thus increase transmission of influenza. We hypothesize that a positive association between rainfall and influenza transmission exists in northern temperate regions, but is unapparent due to the strength and regularity of the cold-weather seasonality patterns of influenza occurrence. Our goal was to study the association between extreme precipitation and emergency room (ER) visits for influenza among people living in a temperate climate. While ample research on specific aspects of weather (rainfall, humidity, temperature, etc.) and influenza transmission/persistence exists with contrary conclusions [16], to our knowledge this is the first investigation of extreme precipitation and influenza ER visits in the Northern US.

## Methods

We conducted a case-crossover study using daily ER visits in the state of Massachusetts (MA) from October 1, 2002 – September 30, 2008. Data were acquired from the State of Massachusetts, Division of Health Care Finance and Policy, Executive Office of Health and Human Services and included patient town of residence, date of visit, age, sex, and the primary and five associated diagnostic codes (International Classification of Disease, Version 9 Clinical Modification (ICD-9-CM)) associated with each visit. Data were acquired solely for administrative purposes, not through interaction with individuals, and contained no identifiable private information. Data were anonymous and determined as data not acquired from human subjects by the U.S. Environmental Protection Agency’s Human Subjects Research Protocol Officer and were therefore considered exempt from Institutional Review Board review. For the purposes of this investigation, only ER visits for influenza, identified as ICD-9-CM code 487, that were listed as the primary cause for ER visits or within the first five associated diagnostic codes were utilized.

### Environmental data

Daily precipitation estimates were generated for each town in MA based upon the National Oceanic and Atmospheric Administration (NOAA)-generated 4 × 4 km grid of daily precipitation estimates during 2002–2008 [17]. Precipitation distributions for each town were estimated using Kriging [18–20] with an external drift based on a simple linear regression defined by elevation. Based on previous research conducted in this study area [21], each town’s extreme precipitation values were defined as precipitation that exceeded the 99th percentile of daily (24-h period starting at 12:00 AM and ending at 11:59 PM) precipitation for that town over the entire study period.

Additional weather variables including air temperature, relative humidity, dew point and barometric pressure were collected from Weather Underground [22], which is a publically available repository for data from the National Weather Service and thousands of weather stations throughout the US. Weather data were included from all 21 weather stations that were in operation during the study period.

The Bayesian space-time downscaling model [23] results for ozone (O_3_) and particulate matter less than 2.5 μm in diameter (PM_2.5_) at each census tract were averaged to create daily values within each town. Ordinary Kriging estimates were calculated for towns without census tracts utilizing data from the other town values.

### Study Design & Data Analysis

The association between extreme precipitation and ER visits for influenza was assessed using a bi-directional case-crossover study design [24]. Under this design, each case functions as their own control at a time point before/after the event of interest (i.e. self-matching), thereby controlling for potential individual confounding factors that do not vary substantially over the course of the case/control selection period (sex, race, socioeconomic status, age). Case days were identified by an ER visit for influenza. Using a time stratified bi-directional approach, control days were defined as days falling on the same day of week and in the same calendar month as the hospital visit (i.e. 3–4 control days per case day). The occurrence of extreme precipitation events was compared between case and control days. This control period selection adjusts for confounding by day of the week, month of the year, and season as well as time-invariant variables (e.g., sex, age, town). It also reduces long-term trends in other time varying confounders by restricting case to control period contrasts to the one month referent window.

Odds ratio (OR) and 95% confidence intervals (CI) for unadjusted and adjusted associations between extreme precipitation and ER visits for influenza were estimated using conditional logistic regression (SAS version 9.4, Cary, North Carolina). Climatological and air quality components O_3_, PM_2.5_, temperature, relative humidity, dew point, and barometric pressure were considered potential confounders and thus included as model covariates. As the effects of temperature on ER visits for influenza may be non-linear, regression splines with degree three polynomials were also assessed when controlling for temperature. We also included a binary indicator of Massachusetts state and federal holidays since they are time-varying but were not able to be matched upon within each month. Multivariable models were selected using stepwise selection and the model with the smallest Akaike information criterion (AIC) was chosen as the best model.

Established variation in influenza susceptibility and incidence by age, gender, race, temperature, and season led us to evaluate these factors as potential effect modifiers of the association between extreme precipitation and influenza ER visits [7, 25, 26]. Effect measure modification on the multiplicative scale was assessed using likelihood ratio tests (*p*-value ≤0.10 as cutoff for statistical significance) to compare models with and without an interaction term. Ages were categorized into four groups (‘0–4’, ‘5–18’, ‘19–64’, ‘65+’ years). A mean daily temperature of 40 °F was utilized as a cutoff under the supposition that snowfall would be unlikely on a day which this threshold was met. Seasons were defined as Winter (December–February), Spring (March–May), Summer (June–August), and Fall (September–November).

No information regarding the length of time between onset of illness and the ER visit was available for individuals included in the study. Since a mean incubation period of 2 days (range 1–4) has been reported for influenza infection [27], we chose to evaluate point estimates for the association between ER influenza visit and an extreme precipitation event within a total lag period ranging from 0 to 6 days, as well as at each individual day lag 0–6. All other weather variables were included in the model utilizing the corresponding lag values.

## Results

**Fig. 1:**
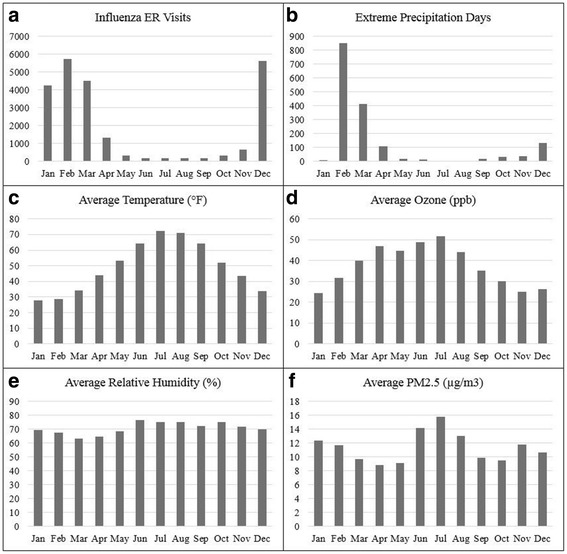
**a** Number of emergency room influenza visits, **b** number of extreme precipitation days, **c** average temperature, **d** average O_3_, **e** average relative humidity, and **f** average PM_2.5_, lag 0–6 days in Massachusetts, by month during October 1, 2002 - September 30, 2008

A total of 23,510 ER visits for influenza occurred in MA during our study period, 1644 (7%) occurred within the 6 days after an extreme precipitation event. Among persons visiting the ER for influenza, 52% (*n* = 12,288) were females and 48% (*n* = 11,220) were males (Table 1). Sixty-five percent of persons visiting the ER for influenza were of white race, 13% were of black race, and 22% were specified as another race; documented information on race is missing for 587 individuals (2.5%). Individuals visiting the ER for influenza ranged in age from 0 to 107 years. Influenza visits peaked in 2003 and again in the year 2008. A seasonal trend for influenza visits was observed with most ER visits occurring during the winter months (Fig. 1a).

**Table 1:** Characteristics of patients admitted to an emergency room for influenza in Massachusetts, October 1, 2002 - September 30, 2008

Influenza (*n* = 23,510)			
Characteristic		Number	Percent
Age in years	0–4	2969	12.63
	5–18	4221	17.95
	19–64	15,036	63.96
	65+	1284	5.46
Gender	Female	12,288	52.27
	Male	11,220	47.72
	Unknown	2	
Race/Ethnicity	White	14,694	65.07
	Black	2881	12.76
	Other	5008	22.18
	Unknown	587	
Year	2002^a^	362	1.54
	2003	6303	26.81
	2004	2761	11.74
	2005	3075	13.08
	2006	3409	14.50
	2007	1840	7.83
	2008^a^	5760	24.50

^a^Partial year. The study period was October 1, 2002 – September 30, 2008

Extreme precipitation values for each town ranged from 1.37–2.08 in./day with an average value of 1.66 in./day over the study period. February, the month with the largest number of days with extreme precipitation also had the highest frequency of influenza emergency room visits (Fig. 1a-b). The daily average temperature ranged from −8 to 89 °F with an average of 46 °F. The average relative humidity was 71% (ranging from 7% to 99%). O_3_ was highly correlated with temperature (Pearson’s *r* = 0.58, *p* < .0001) and levels ranged from 0.75 to 129 ppb (average 37 ppb). Daily PM_2.5_ ranged from <1 μg/m^3^ to 48 μg/m^3^ with an average of 11 μg/m^3^. Temperature, O_3_, and PM_2.5_, values peaked in summer months; relative humidity revealed no statistically significant difference by season (Fig. 1c-f).

**Fig. 2:**
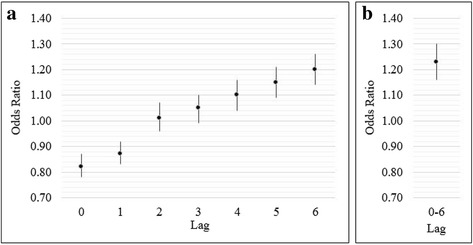
Analysis comparing adjusted odds ratio (95% confidence interval) effects of influenza emergency room visits associated with extreme precipitation (≥99th percentile) at **a** individual lag days 0–6 and **b** cumulative lag days 0–6

Crude analysis revealed that extreme precipitation, occurring during our pre-specified lag of 0–6 days, was significantly associated with ER visits for influenza (OR = 1.13 (95%CI: 1.07, 1.20)). The best model, determined by the smallest AIC, included O_3_, PM_2.5_, humidity, holidays, and a cubic spline for temperature, and resulted in an adjusted OR of 1.23 (95%CI: 1.16, 1.30). Using this same multivariable model to explore the effects of extreme precipitation at individual lag days 0, 1, 2, 3, 4, 5, and 6 (Fig. 2), we observed an inverse association between extreme precipitation and influenza ER visits at lags 0 and 1 and no statistically significant association at lags 2 and 3. Among individual lags 4, 5, and 6 each resulted in significant positive associations between extreme precipitation and ER visits for influenza, with day 6 producing the highest effect estimate (OR = 1.20 (95%CI: 1.14, 1.26)).

The effect of extreme precipitation on ER visits for influenza was modified by race (*p* = 0.035; Table 2). The association among Blacks (OR = 1.48 (95%CI: 1.30, 1.68)) was higher than the observed association among other races. The smallest effect was observed among individuals identified as ‘Other’ race. Extreme precipitation was significantly associated with ER visits for influenza in children ages 5–18 years (OR = 1.32 (95%CI: 1.14, 1.52) and adults ages 19–64 years (OR = 1.23 (95%CI: 1.15, 1.32). Odds ratios in pre-school age children (ages 0–4) and the elderly (age 65+) were positive, but did not reach statistical significance. Likelihood ratio tests revealed no significant effect modification across gender, temperature, or season.

**Table 2:** Adjusted odds ratios of influenza emergency room visits associated with extreme precipitation (≥99th percentile) within 0–6 days, stratified by individual level characteristics

	OR (95% CI)	*p*
Age group		
0–4	1.12 (0.93, 1.35)	
5–18	1.32 (1.14, 1.52)	
19–64	1.23 (1.15, 1.32)	
65+	1.11 (0.85, 1.45)	0.398
Gender		
Male	1.23 (1.13, 1.34)	
Female	1.23 (1.13, 1.33)	0.987
Race		
White	1.24 (1.04, 1.48)	
Black	1.48 (1.30, 1.68)	
Other	1.18 (1.10, 1.27)	0.035
Temperature		
< 40 °F	1.20 (1.12, 1.28)	
≥ 40 °F	1.38 (1.21, 1.59)	0.156
Season		
Winter (Dec-Feb)	1.22 (1.13, 1.32)	
Spring (Mar-May)	1.23 (1.11, 1.36)	
Summer (Jun-Aug)	1.18 (0.69, 1.99)	
Fall (Sept-Nov)	1.31 (1.00, 1.72)	0.466

## Discussion

To our knowledge this case-crossover study was the first to investigate the association between the occurrence of extreme precipitation events and ER visits for influenza in a northern temperate climate. Despite the fact that many people do not actually go to the ER (or even the doctor) for the flu, we observed an increased risk of ER visits for influenza after extreme precipitation events among Massachusetts residents during 2002–2008. This association was significantly higher among Blacks. Occurrence of influenza visits to the ER followed the temperate zone pattern of seasonal influenza occurrence observed in the U.S. each year with ER visits peaking in the winter months.

Research previously conducted exploring the effects of various climatic factors, including precipitation and humidity, on influenza transmission and persistence, have yielded conflicting results. For example, while studies exist showing influenza epidemics associated with high specific humidity in the tropics [28, 29], reports of enhanced influenza transmission during periods of low ambient humidity have also been described. Inconsistent findings likely result from variations in study locale and climate, additional weather conditions incorporated in the analysis, influenza case definitions, and other socio-demographic variables examined.

Our study found a significant positive association between extreme precipitation and ER visits for influenza over the 0–6 day lag period. In addition, results from the multivariable analyses of individual lags are consistent with the known epidemiology of influenza. We observed negative associations of extreme precipitation events with ER visits for influenza at lags 0–1 and positive associations at lags 2–6. We considered two potential reasons for this observation, 1) the negative effect estimates observed may be indicative of the fact that extreme weather events often impede access to care preventing individuals from receiving medical treatment [30, 31]. If individuals in this study population were less likely to seek emergency medical care during periods of extreme precipitation, this could account for the observed significant inverse association between extreme precipitation and ER visits for influenza at lag 0. And 2) the absence of a significant positive association between extreme precipitation and influenza ER visits at individual lags 0–3 is consistent with the established influenza incubation period and usual onset of symptoms, generally observed at approximately 1–4 days (average of 2 days) post infection [27]. Indeed, point estimates rise at lag day three and achieve significance at lag day 4, consistent with a course of illness lasting a day on average before a patient initiated an ER visit.

During the study period, Blacks visiting the emergency room visits for influenza (12%) were overrepresented relative to their proportion of the Massachusetts state population (7%) [32]. This observation is consistent with existing literature, which show Blacks in the US overrepresented for influenza ER visits [33] and having more than twice the rate of overall ER visits than Whites [34]. The relationship between extreme precipitation and ER visits for influenza was modified by race with the association being significantly higher among Blacks. Racial or ethnic barriers to widespread influenza vaccination may be present [35, 36], which may increase vulnerability to influenza and affect the likelihood of an individual eventually visiting the ER. Other studies have also suggested the presence of differential host susceptibility such as socioeconomic vulnerabilities and potentially lower serum vitamin D concentrations among African Americans [37, 38], factors that could lead to more ER visits for influenza among this population, but were unavailable for assessment within this study.

There was no statistically significant effect modification by age. It should be noted that while positive but non-significant point estimates were observed for the association between extreme precipitation and ER visits for influenza among young children (0–4) and the elderly (65+), individuals within these age groups would be most likely to go (or be taken) to the ER for the influenza. Point estimates for ages 5–64 years were both higher (than the very young and elderly) and statistically significant, a possible result from sample size differences between age groups. When stratified by age, children 5–18 years experienced the highest risk of ER visits for influenza; this may be reflective of congregation in indoor school settings, which often temporally coincides with increased reports of influenza [39, 40].

Crowding, a known risk factor for influenza [9, 41], is one plausible explanation for the observed association between extreme precipitation events and ER visits for influenza as extreme precipitation would likely results in individuals congregating indoors irrespective of outside temperatures. Additional time spent indoors in closer contact with other household members during the cold, winter season in temperate climates and the rainy season in tropical climates may provide more opportunities for person-to-person spread of acute respiratory pathogens via close contact. A previously conducted case-crossover analysis in Dhaka [9] revealed an association between rain and acute respiratory infections, including influenza. Though indoor congregation and crowding may be contributing factors, amplifying an otherwise less-pronounced change in transmission due to extreme precipitation [7], we did not have access to individual-level data within each household. Future investigations to elucidate these characteristics are warranted.

While indoor congregation is a plausible and reasonable potential mechanism to explain the observed association between extreme precipitation and ER visits for influenza in our study, it is also possible that microbes act somewhat differently during heavy downpours. For instance, wet conditions of a tropical climate (or extreme precipitation) may allow the influenza virus to adhere to more surfaces within a room. Though unable to survive in the air so well, the influenza virus could instead be thriving on fomites, making it more likely to be transmitted from contaminated indoor surfaces.

A major strength of this study is the time stratified bi-directional case-crossover design which used the same day of the week in the referent month for control period selection. This ensures that factors which are not time varying such as individual characteristics (age, race, gender) are controlled for automatically [42]. Additionally, this method safeguards against confounding by strong seasonal effects through matching within calendar month. Factors that did vary with time were assessed as confounders in the analysis. For example, holidays which are time-varying, can potentially affect rates of hospitalization, and may reduce influenza transmission, were included in the final model. We also created precipitation estimates for every town which allowed us to assign exposure status for each individual resident’s visit to the ER for influenza.

This investigation was limited in its ability to distinguish among varying types of precipitation (i.e. snow, freezing rain, rain) present during the study period. It is possible that the effects of extreme snowfall on emergency room visits for influenza would differ from the effects of extreme rainfall. However, we tested for effect modification by temperature and season, which are both related to precipitation type, and found no evidence for effect modification. Potential effect modification by precipitation rates (flash flooding vs. steady precipitation) should also be explored in future analyses where possible.

The use of hospital administrative data, specifically ICD-9 diagnostic codes, means we cannot rule out the possibility that some patients presented influenza like illness and were not actually tested for infection with influenza virus, however this misclassification is likely nondifferential with respect to exposure status. Additionally, emergency department data is often used for influenza syndromic surveillance, acting as a general indicator of influenza morbidity in the target population [43].

## Conclusion

To our knowledge this study was the first to investigate the association between extreme precipitation and ER visits for influenza in a Northern temperate climate. The results from this case-crossover analysis reveal a positive association between extreme precipitation events and increased ER visits for influenza. As extreme precipitation is expected to increase in frequency in the Northeastern U.S. during climate change [44], investigations into the range and magnitude of potential health effects associated with these climate-change associated events are essential for estimating the economic and social costs of uncontrolled climate change. Such work has the potential to guide societal adaptation to changing weather and environmental conditions such as implementing influenza prevention strategies within shelters housing large numbers of people following disaster events outside of the traditional influenza season, especially during pandemic years. Considering the impacts of extreme weather, additional research focused on extreme precipitation events and influenza is warranted.

## Abbreviations

AIC: Akaike information criterion
CI: Confidence intervals
ER: Emergency room
ICD-9-CM: International Classification of Disease, Version 9 Clinical Modification
MA: Massachusetts
NOAA: National Oceanic and Atmospheric Administration
O_3_: Ozone
OR: Odds ratio
PM_2.5_: Particulate matter less than 2.5 μm in diameter
